# Ebulin l Is Internalized in Cells by Both Clathrin-Dependent and -Independent Mechanisms and Does Not Require Clathrin or Dynamin for Intoxication

**DOI:** 10.3390/toxins13020102

**Published:** 2021-01-30

**Authors:** Rosario Iglesias, José M. Ferreras, Alicia Llorente, Lucía Citores

**Affiliations:** 1 Department of Biochemistry and Molecular Biology and Physiology, Faculty of Sciences, University of Valladolid, E-47011 Valladolid, Spain; riglesia@bio.uva.es (R.I.); josemiguel.ferreras@uva.es (J.M.F.); 2 Department of Molecular Cell Biology, Institute for Cancer Research, Oslo University Hospital, 0379 Oslo, Norway; alillo@rr-research.no; 3 Department of Mechanical, Electronics and Chemical Engineering Art and Design, Oslo Metropolitan University, 0130 Oslo, Norway

**Keywords:** apoptosis, clathrin, dynamin, ebulin, endocytosis, intracellular transport, lectin, rRNA *N*-glycosylase, ribosome-inactivating protein, ricin

## Abstract

Ebulin l is an A-B toxin, and despite the presence of a B chain, this toxin displays much less toxicity to cells than the potent A-B toxin ricin. Here, we studied the binding, mechanisms of endocytosis, and intracellular pathway followed by ebulin l and compared it with ricin. COS-1 cells and HeLa cells with inducible synthesis of a mutant dynamin (K44A) were used in this study. The transport of these toxins was measured using radioactively or fluorescently labeled toxins. The data show that ebulin l binds to cells to a lesser extent than ricin. Moreover, the expression of mutant dynamin does not affect the endocytosis, degradation, or toxicity of ebulin l. However, the inhibition of clathrin-coated pit formation by acidification of the cytosol reduced ebulin l endocytosis but not toxicity. Remarkably, unlike ricin, ebulin l is not transported through the Golgi apparatus to intoxicate the cells and ebulin l induces apoptosis as the predominant cell death mechanism. Therefore, after binding to cells, ebulin l is taken up by clathrin-dependent and -independent endocytosis into the endosomal/lysosomal system, but there is no apparent role for clathrin and dynamin in productive intracellular routing leading to intoxication.

## 1. Introduction

Ribosome inactivating proteins (RIPs) are a family of well-characterized toxins that specifically and irreversibly inhibit protein synthesis. RIPs belong to a class of enzymes (EC 3.2.2.22) that exhibits rRNA N-glycosylase activity. This activity prevents protein synthesis by causing the release of a specific adenine residue in the sarcin-ricin loop (SRL) of the large rRNA that is crucial for interaction of the elongation factor with the ribosome [1].

Most RIPs are produced by plants, where they may play a role in defense mechanism against predators, fungi, and viruses [2,3]. RIPs also show toxicity towards animal cells, targeting the host protein synthesis machinery. In addition to rRNA damage, RIPs can induce apoptosis [4,5].

There are important differences in toxicity among RIPs depending on their ability to reach the ribosomes in the cytosol of target cells. Since RIPs are unable to cross the plasma membrane directly, they use existing cellular mechanisms designed for uptake of macromolecules. Following initial internalization, RIPs are transported within the cell to the particular membrane where toxin translocation to the cytosol occurs. Type 1 RIPs consisting of a single enzymatic active (A) chain often display lower toxicity than type 2 RIPs which consist of a binding (B) chain with lectin activity linked by a disulfide bond to the enzymatic A chain. The carbohydrate-binding domains of the B chain recognize glycosylated receptors on the cell surface, facilitating the entry of the A chain into the cell [5]. However, the presence of the B chain is not sufficient to confer a high level of cytotoxicity on all type 2 RIPs. Based on their toxicity to mammals, type 2 RIPs are divided into two groups: the toxic and nontoxic type 2 RIPs [6]. The former group includes ricin, abrin, viscumin, volkesin, and stenodactylin, which are among the most potent plant toxins. In contrast, ebulin l, nigrin b, *Ricinus* agglutinin (RCA), *Iris* agglutinin (IRA) b/r, and cinnamomin belonging to the latter group show little or no toxicity in higher animals. The reason for the different toxicities among type 2 RIPs is not clear. It could rather be attributed to differences between the B chains, which are responsible for the interaction with cellular membranes, than to the enzymatic A chains, which inactivate naked ribosomes with apparently similar efficiency.

Ricin, a toxin isolated from *Ricinus communis* L., is the archetype of the toxic type 2 RIP family. The structure, biochemistry, and cytotoxicity of this 64-kD A-B toxin have been extensively examined and reviewed [7,8,9,10]. In order to enter and intoxicate cells, ricin first has to bind to cell surface receptors. Ricin binds to both glycoproteins and glycolipids with terminal galactose and then is internalized by different endocytic mechanisms. After being endocytosed, most of the ricin molecules are either recycled or transported to lysosomes for degradation. However, a small proportion (5%) of ricin is transported to the Golgi apparatus and then retrogradely to the endoplasmic reticulum (ER). After a reduction of the internal disulfide bond that connects the A and B chain, the A chain enters the cytosol using the quality control pathway that leads to ER-associated protein degradation (ERAD). Once in the cytosol, a small fraction of the toxin is able to escape ubiquitination and degradation by the proteasome and binds to its ribosomal target [7,9].

In recent years, an extensive study for the presence of RIPs in several species of the genus *Sambucus* has allowed the isolation of more than 20 toxins. All of the type 2 RIPs found in the genus *Sambucus* are considered nontoxic type 2 RIPs since, despite being as toxic as ricin at the ribosomal level, they display much less toxicity to cells and animals. Nontoxic type 2 RIPs specific for galactose [11,12], tetrameric type 2 RIPs specific for sialic acid [13,14], nontoxic type 2 RIPs lacking sugar binding activity [15,16], and nontoxic type 2 RIPs with affinity for N-acetyl-glucosamine oligomers [17] have been described for the first time in the genus *Sambucus*.

Ebulin l, a 56 kD A-B toxin obtained from the leaves of *Sambucus ebulus* L., was one of the first nontoxic type 2 RIPs isolated [11]. The structure of ebulin l has been resolved by X-ray diffraction analysis, and the tertiary structure closely resembles that of ricin [18]. In the A chain, ebulin l has roughly the same positioning of key active site residues as ricin. This is consistent with the fact that both proteins have a similar inhibitory activity of protein synthesis in cell-free systems. The overall fold of the ebulin and ricin B chains is very similar. However, ebulin l has a lower affinity for galactose than ricin due to a change in the structure of the 2-γ subdomain of the ebulin B chain. In fact, it was found that ebulin l has different binding properties to D-galactose-containing matrixes than ricin [16,18]. This reduced affinity for galactosides could alter the ability of the B chain to bind cells and could affect the uptake and the intracellular fate of the toxin. In contrast to the high enzymatic activity on ribosomes, the toxicity of ebulin l on animal cells was found to be about 10^4^–10^6^ times lower than the toxicity of ricin [11,16]. In mice, the LD_50_ of ebulin l administered by intraperitoneal injection is 2 mg/kg body weight, while for ricin, it is in the range of a few micrograms per kilogram [11].

RIPs are potent inhibitors of protein synthesis that have been used for the construction of conjugates and immunotoxins [5,19]. Linked to a targeting portion such as an antibody or a protein that specifically binds to a receptor, toxins have been used to specifically kill tumor cells. Ebulin l has been used in different conjugates and immunotoxins targeting tumor cells with high selectivity [20]. The main advantage of ebulin l over ricin and its derivatives is its reduced cytotoxicity. Antibodies or ligands led the internalization and promoted the productive translocation of ebulin l to the cytosol, thus allowing for its anti-ribosomal activity. To improve the efficiency of selective targeting of ebulin l to malignant cells, a better understanding of endocytosis and the cellular transport and toxicity mechanisms of ebulin l is essential. However, very little is known about the receptors that mediate the cellular uptake of ebulin l or its intracellular transport. Therefore, in this work, the binding, the mechanism of endocytosis, and the intracellular pathway followed by the nontoxic type 2 RIP ebulin l were investigated. Moreover, the transport of ebulin l and the toxic type 2 RIP ricin were compared. To investigate the mechanism of ebulin l internalization, we used HeLa cells with inducible expression of a mutant dynamin (K44A) that blocks clathrin-dependent and some clathrin-independent pathways (caveolae, RhoA, fast endophilin-mediated endocytosis (FEME), and others). In addition, cytosol acidification was also used to inhibit endocytosis from coated vesicles in COS and HeLa dynK44A cells. Our results show that, after binding to the cells, ebulin l is taken up by clathrin-dependent and -independent endocytosis into the endosomal/lysosomal system, but there is no apparent role for clathrin and dynamin in productive intracellular routing leading to intoxication.

## 2. Results and Discussion

### 2.1. Binding, Endocytosis, Recycling, and Degradation of Ebulin l and Ricin in COS Cells

#### 2.1.1. Binding

**Figure 1:**
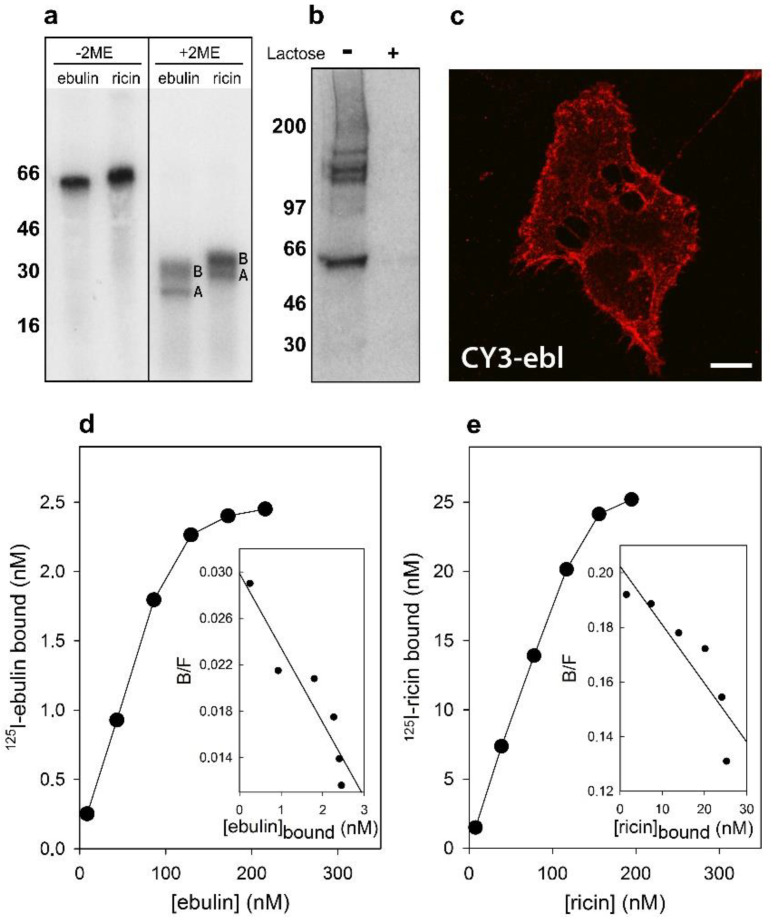
Binding of ebulin l and ricin to COS cells: (**a**) the ^125^I-labeled ebulin l and ^125^I-labeled ricin were analyzed by sodium dodecyl sulphate–polyacrylamide gel electrophoresis (SDS-PAGE) either in the absence or the presence of 2-mercaptoethanol (2ME) followed by autoradiography. A and B indicate the corresponding A and B chains of the toxins. Molecular weight standards (kD) are indicated on the left of the gels. (**b**) Crosslinking of bound ^125^I-labeled ebulin l to COS cells: ^125^I-ebulin l was added to the cells for 1 h at 4 °C in the presence or absence of 0.1 M lactose. Then, the cells were treated for 20 min at 4 °C with 0.3 mM disuccinimidyl suberate to induce crosslinking and were analyzed by SDS-PAGE and autoradiography. (**c**) Binding of fluorescent ebulin l to COS cells: The cells were incubated at 4 °C for 1 h with CY3-ebulin l (CY3-ebl) to allow binding and then fixed immediately. Bar, 50 µm. (**d**,**e**) Toxin binding and Scatchard plots: the binding of ^125^I-ebulin l and ^125^I-ricin to cells was measured by adding increasing concentrations of the labeled toxin to cells at 4 °C. After 1 h, any unbound toxin was removed by washing and the amount of radioactivity associated with the cells was measured. The insets are Scatchard plots of the binding data. These experiments were repeated twice with similar results. (B/F) Bound/Free.

Ebulin l and ricin are A-B toxins consisting of an enzymatic A chain with rRNA N-glycosylase activity linked by a disulfide bond to a binding B chain with lectin activity. Both RIPs are galactose-binding lectins [16,18] and therefore bind to different molecules with terminal galactose on the cell surface. To test for specific binding to COS cells, toxins labeled with radioactive iodine (Figure 1a) as well as fluorescent labeled ebulin l were used.

Crosslinking experiments after preincubation of the cells for 1 h at 4 °C with ^125^I-labeled ebulin l demonstrated that there are receptors for ebulin l at the surface of these cells. In addition to labeled toxin, bands migrating higher than 100 kD were observed (Figure 1b). As expected, the addition of 0.1 M lactose to parallel cultures incubated in the same conditions prevented crosslinking of ^125^I-ebulin l to the receptors (Figure 1b). Moreover, when the cells were treated with CY3-ebulin l at 4 °C, the toxin was bound to the cell surface in a homogenous manner (Figure 1c). To better quantify the number of receptors for ebulin l and ricin, COS cells were incubated with increasing concentrations of the labeled toxins at 4 °C. The binding experiments showed that ^125^I-ebulin l was bound in a saturable way to COS cells with a K_d_ of 1.5 × 10^−7^ M and 2.8 × 10^6^ binding sites per cell (Figure 1d), whereas for ^125^I-ricin, a K_d_ value of 4.6 × 10^−7^ M and 5.6 × 10^7^ binding sites per cell were measured (Figure 1e). The binding affinity determined by the K_d_ values was low and comparable for the two toxins. It is believed that 10^6^–10^8^ ricin molecules can be bound to the cell surface [9]. Thus, we found that the number of cell receptors for ebulin l was 20 and 150 times lower than that for ricin in COS and HeLa dynK44A cells (see Section 2.2), respectively. It has been shown that ebulin l has a lower affinity for galactose than ricin due to a change in the structural disposition of the 2γ-subdomain of the ebulin B chain, which limits its ability to bind galactosides on cell surfaces [18]. This may explain the differences in the number of cell surface receptors observed. For ricin, it is likely that the binding to many different receptors results in multiple intracellular transport pathways that could deliver ricin to the appropriate compartment for membrane translocation to the cytosol. It is then possible that the differential affinity of ebulin l for galactosides determines its intracellular fate and possibly its cytotoxicity. However, differences in binding (20–150 times higher for ricin) cannot explain the lower cytotoxicity of ebulin l compared to ricin (10^5^ times higher for ricin). Accordingly, it has been shown that HeLa cells have a similar number of receptors for the nontoxic RIP nigrin b as that for modeccin and volkensin, which are more toxic than ricin, and two-log lower receptor numbers than for ricin [21]. It has also been shown that cinnamomin, a nontoxic type 2 RIP, and ricin share similar binding sites on BA/F3β cells with different affinity and that the lower cytotoxicity of cinnamomin is due to its B-chain [22]. However, the type 2 RIP articulatin-D, which lacks lectin activity, has been shown to display a cytotoxicity comparable to that of highly toxic type 2 RIPs, indicating that, for uptake and subsequent toxicity of all type 2 RIPs, recognition by the B chain of glycosylated receptors on the cell surface may not be essential [23].

#### 2.1.2. Endocytosis, Recycling, and Degradation

**Figure 2:**
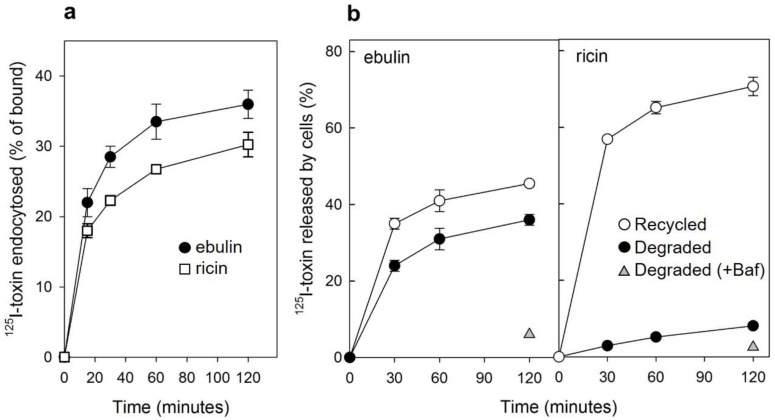
(**a**) Kinetics of ebulin l (circles) or ricin (squares) internalization in COS cells: the cells were incubated with ^125^I-ebulin or ^125^I-ricin at 37 °C for 0 to 120 min and the amount of bound and endocytosed toxins were quantified as described in Section 4.2.2. The data are expressed as the internalized radioactivity in percentage of the total radioactivity associated with the cells. (**b**) Recycling and degradation of ebulin l and ricin in COS cells: the cells were incubated with ^125^I-ebulin l or ^125^I-ricin for 20 min at 37 °C. Surface-bound toxins were removed by 0.1 M lactose and the incubation continued for the times indicated. Recycling (open circles) was measured as the amount of trichloroacetic acid (TCA)-precipitable toxin in the medium and at the cell surface. Degradation (closed circles) was measured as the amount of radioactivity that could not be precipitated by TCA. Degradation in the presence of bafilomycin A1 (Baf) was measured after 120 min (triangles). In both cases, the data are expressed as a percent of the total radioactivity. The data represent the mean ± SD of two experiments.

Ebulin l has been shown to be internalized upon binding to glycoproteins and glycolipids containing terminal galactose [16]. To study this process further, COS cells were incubated with ^125^I-labeled ebulin l or ricin at 37 °C for different periods of time. Considering that lactose removes surface-bound toxins but not internalized toxins, toxin internalization was measured as the ratio of endocytosed to surface-bound ^125^I-labeled toxin at different time points. As shown in Figure 2a, the amount of internalized ebulin l and ricin levelled off after 30 min, indicating that toxin uptake and intracellular processing approached equilibrium at this time. Approximately 28% of total cell-associated ^125^I-ebulin l and 23% of total cell-associated ^125^I-ricin were internalized into the COS cells during a 30 min incubation period at 37 °C.

We next studied the ability of COS cells to recycle internalized toxins back to the surface and to the culture medium. As shown in Figure 2b, recycling of toxins, measured as trichloroacetic acid (TCA)-insoluble radioactivity in the medium, occurred as a rapid phase that lasted for less than 30 min, followed by a slower phase. Approximately 45% of ebulin l and 70% of ricin were found in the TCA-insoluble fraction after 2 h of incubation at 37 °C. We also measured the degradation of toxins internalized by COS cells. As shown in Figure 2b, only 8% of the total internalized ^125^I-ricin was found to be degraded in COS cells after 2 h incubation at 37 °C. However, a higher percentage of ebulin l (36%) was found in the TCA-soluble fraction. Bafilomycin A1, an inhibitor of the vacuolar H^+^-ATPase [24], inhibited the degradation of ebulin l and ricin, indicating that this process takes place in an acidic compartment. According to this, nigrin b, a nontoxic type 2 RIP, was shown to enter HeLa cells in a similar way to ricin; however, it was much faster and widely degraded. Moreover, the nigrin b-derived material released by cells was completely inactive [21,25].

#### 2.1.3. Mechanism of Endocytosis

**Figure 3:**
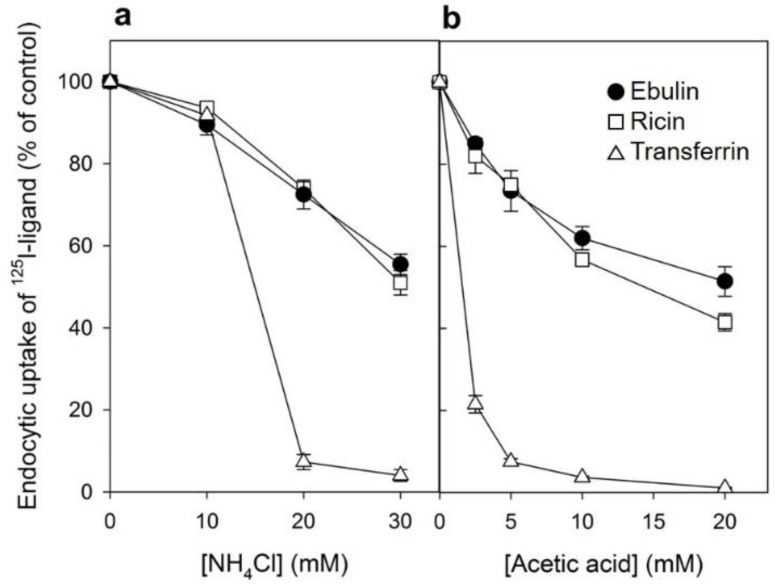
Effect of acidification of the cytosol on the ability of COS cells to internalize ^125^I-ebulin l, ^125^I-ricin, and ^125^I-transferrin: (**a**) COS cells were incubated for 30 min at 37 °C in 4-(2-hydroxyethyl)-1-piperazineethanesulfonic acid (HEPES) medium pH 7 with the indicated concentrations of NH_4_Cl. The medium was removed, and a solution containing 0.14 M KCl, 2 mM CaCl_2_, 1 mM amiloride, 1 mM MgCl_2_, and 20 mM HEPES, pH 7.0, was added. After 5 min of incubation at 37 °C, ^125^I-ebulin l, ^125^I-ricin, or ^125^I-transferrin were added, and cell bound and endocytosed proteins were measured after 20 min of incubation for ebulin l and ricin and after 5 min for transferrin. Symbols: ●, ebulin l; ☐, ricin; and △, transferrin. (**b**) The cells were incubated for 5 min at 37 °C in HEPES medium, pH 5.5, with increasing concentrations of acetic acid. ^125^I-ebulin l, ^125^I-ricin, or ^125^I-transferrin were then added, and after 20 min of incubation for ebulin l and ricin and after 5 min for transferrin, the amount of endocytosed proteins was measured as described above. Symbols: ●, ebulin l; ☐, ricin; and △, transferrin. The data represent the mean ± SD of two experiments.

Several endocytic mechanisms have been documented, including macropinocytosis, clathrin-dependent endocytosis, caveolae-dependent endocytosis, and clathrin- and caveolae-independent endocytosis [26]. It has been demonstrated that ricin is able to employ different endocytic mechanisms, probably because it can recognize and bind to a great variety of cell surface components [9]. Clathrin-independent endocytosis was first described by studying the uptake of ricin, which continued after the inhibition of clathrin-dependent endocytosis [27]. To determine if ebulin l and ricin are internalized from clathrin-coated pits in COS cells, the cytosol was acidified to inhibit internalization from clathrin-coated pits [27]. When the cytosolic pH falls below 6.5, clathrin-coated pits at the cell surface can no longer pinch off and form clathrin-coated vesicles. In this study, the cytosol was acidified by preloading the cells with increasing concentrations of NH_4_Cl followed by its removal [27]. The data in Figure 3a show that, in COS cells, the uptake of ebulin l and ricin were reduced by about 50% after acidification of the cytosol. Control experiments showed that the uptake of transferrin, which is endocytosed by clathrin-dependent endocytosis [28], was reduced by more than 95% under the same conditions. When the cytosol of COS cells was acidified by incubation with acetic acid, similar results to that with NH_4_Cl pre-pulsing were obtained. Thus, while the endocytosis of transferrin was strongly reduced, there was only an approximately 50% and 55% reduction in the uptake of ^125^I-ebulin and ^125^I-ricin, respectively (Figure 3b). This indicates that ebulin l and ricin uptake is mediated by both clathrin-dependent and clathrin-independent endocytosis in COS cells.

#### 2.1.4. Intracellular Transport of Ebulin l in COS Cells

**Figure 4:**
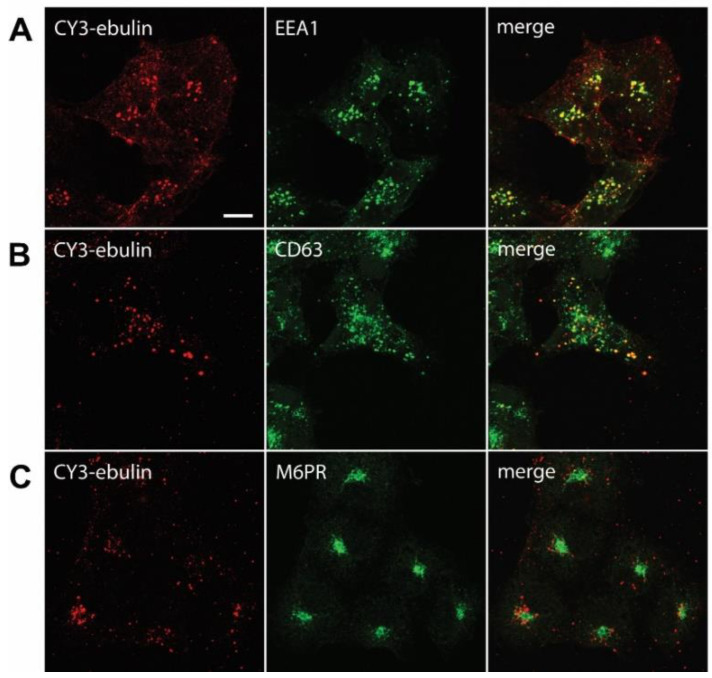
Transport of fluorescent ebulin l in COS cells and colocalization with markers for different intracellular organelles: the cells were incubated at 4 °C for 1 h with CY3-ebulin l to allow binding of the protein and then incubated at 37 °C for 10 min (**A**) and 60 min (**B** and **C**) before fixation. Then, the cells were stained with anti-EEA1 (early endosomes) (**A**), anti-CD63 (late endosomes/lysosomes) (**B**), and anti M6PR (Golgi) (**C**) antibodies followed by fluorescein isothiocyanate (FITC)-conjugated secondary antibody. Colocalizations between CY3-ebulin and the different markers were quantified using Pearson’s correlation coefficient (PCC). PCC between CY3-ebulin and EEA1 = 0.42 ± 0.08; PCC between CY3-ebulin and CD63 = 0.24 ± 0.07; and PCC between CY3-ebulin and M6PR = −0.05 ± 0.03. The PCC values represent the mean ± SD of 5 images analyzed for each marker. Bar, 50 µm.

After endocytosis, RIPs move within the endosomal system until they reach the appropriate compartment for entry into the cytosol, where they inactivate ribosomes. To determine the intracellular transport of ebulin l in COS cells, ebulin l was labeled with the fluorophore CY3 and incubated with COS cells. The cellular distribution of ebulin l was studied after incubation at 37 °C for various periods of time. The data in Figure 1c demonstrate that, when the cells were treated with ebulin l at 4 °C, the toxin is evenly bound all over the cell surface. When the cells were subsequently incubated at 37 °C, the amount of ebulin l at the surface was reduced and the fluorescent ebulin l appeared as intracellular dots, suggesting uptake in vesicles (Figure 4). We next performed double-labeling experiments with EEA1 (early endosome antigen 1), a protein that is associated with early endosomes [29]. As shown in Figure 4A, incubation for 10 min at 37 °C resulted in good colocalization of EEA1 and the ebulin l-labeled intracellular structures (Pearson’s correlation coefficient (PCC) = 0.42). After 60 min at 37 °C, ebulin l remained in vesicles but did not colocalize to a large extent with EEA1 (data not shown). Instead, some of the structures stained positive for CD63, a late endosome/lysosome marker, colocalized with ebulin l (PCC = 0.24), indicating that the toxin is transported to lysosomes for degradation (Figure 4B). Moreover, ebulin l did not colocalize with mannose-6-phosphate receptor (M6PR), a marker for late endosomes and the trans-Golgi network [30] (PCC = −0.05) (Figure 4C). There is a possibility that only a very small fraction of ebulin l is transported to the Golgi apparatus and that this was not detectable in our assay. However, these results together with further experiments (see below) suggest binding and entry of ebulin l into the endosomal/lysosomal compartment but not to the Golgi apparatus.

**Figure 5:**
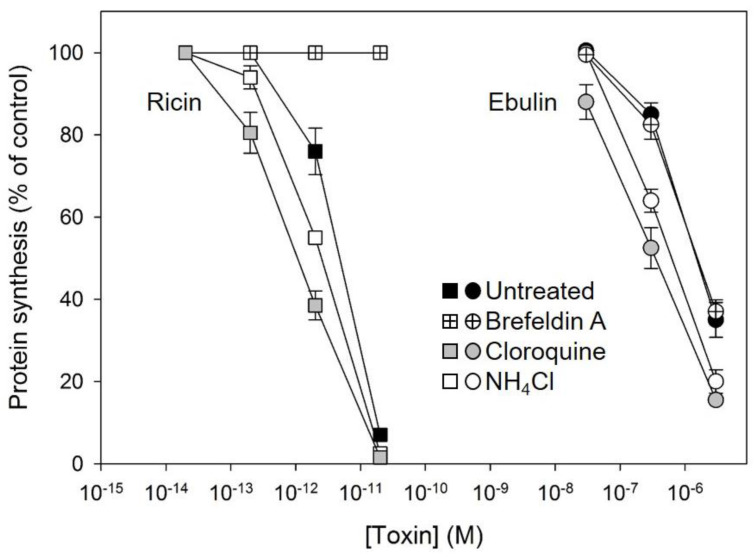
Effect of brefeldin A, chloroquine, and NH_4_Cl on protein synthesis in COS cells treated with ebulin l (circles) and ricin (squares): the cells were left untreated (black symbols) or preincubated with 5 µg/mL brefeldin A (crossed symbols), 25 μM cloroquine (grey symbols), and 20 mM NH_4_Cl (open symbols) for 1 h and then incubated with different concentrations of ebulin l and ricin for 18 h. Protein synthesis was finally measured as indicated in Section 4.2.4. The data represent the mean ± SD of two experiments performed in duplicate.

Some bacterial toxins, such as diphtheria toxin and anthrax toxin, are translocated to the cytosol from acidic endosomes [31,32]. By contrast, ricin and other bacterial toxins such as cholera toxin and Shiga toxin follow the retrograde pathway from endosomes to the Golgi complex and further to the endoplasmic reticulum before the A chain is translocated to the cytosol [33]. To better understand how ebulin l enters the cytosol, we studied the effects of agents interfering with intracellular routing on the cytotoxic process. Lysosomotrophic amines such as NH_4_Cl and chloroquine raise the pH within acidic intracellular vesicles. Preincubation of the COS cells with chloroquine and NH_4_Cl enhanced the cytotoxicity of ebulin l as well as that of ricin (Figure 5). This indicates that ebulin l, similar to ricin, does not require a low pH for translocation to the cytosol. Moreover, the lysosomotrophic amines may stimulate cytotoxicity by preventing toxin degradation by inactivating the lysosomal enzymes, possibly due to an increase in the intralysosomal pH. In addition, we investigated the effect of the fungal inhibitor brefeldin A, which causes Golgi complex disassembly and has been shown to inhibit ricin toxicity. As shown in Figure 5, preincubation of COS cells with brefeldin A markedly reduced as expected the cytotoxicity of ricin but had no significant effect on the cytotoxicity of ebulin l (Figure 5). This indicates that ebulin l follows a Golgi-independent pathway to the cytosol.

### 2.2. Binding, Endocytosis, and Degradation of Ebulin l and Ricin in HeLa Cells Overexpressing dynK44A

As shown in Figure 3, in COS cells, ebulin l, similar to ricin, was still endocytosed when the formation of clathrin-coated vesicles was inhibited by acidification of the cytosol. This suggests that clathrin-independent endocytosis is responsible for approximately 50% of the ebulin l uptake in those cells. In order to investigate further the mechanism of ebulin l internalization, we used HeLa dynK44A cells, which are HeLa cells with inducible synthesis of a mutant dynamin (K44A) [34]. It has been reported that the GTPase dynamin mediates the scission of clathrin-coated pits and that it is also involved in the budding of caveolae. It has been shown that, in those cells, ricin is internalized by clathrin- and caveolae-independent endocytosis [35]. HeLa dynK44A cells express, under tetracycline regulation, the dominant negative dynamin K44A mutant unable to bind and hydrolyze GTP. When the mutant dynamin is induced by the removal of tetracycline for two days, the cells are defective in clathrin-mediated endocytosis as well as in endocytosis from caveolae [34,36,37]. It has been shown that the prolonged inhibition of clathrin-dependent endocytosis in HeLa dynK44A allows the induction of compensatory mechanisms activating clathrin-independent endocytosis [35,38].

First, we studied the binding of ^125^I-ebulin l and ^125^I-ricin and found that HeLa dynK44A cells bound approximately 150 times more ricin than ebulin l. Scatchard analysis indicated that the number of binding sites for ebulin l was approximately 2.5 × 10^5^ and 3.7 × 10^7^ for ricin (Figure S1). Cells bound approximately the same amount of ^125^I-ebulin l independently of mutant expression (data not shown).

**Figure 6:**
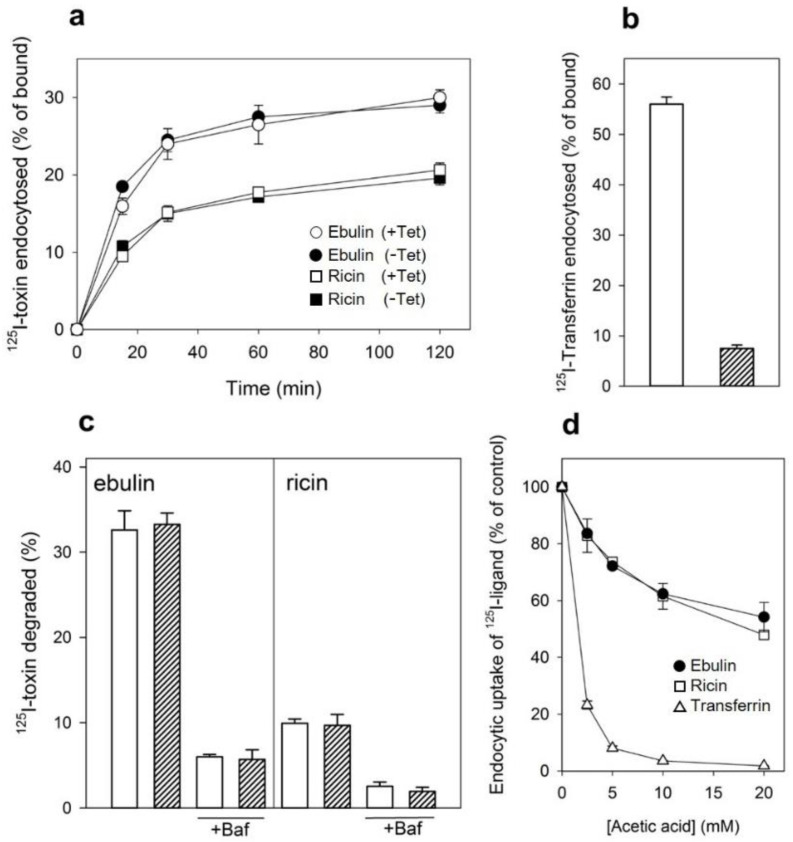
(**a**) Rate of internalization of ebulin l (circles) or ricin (squares) in HeLa dynK44A cells, with (closed symbols) and without (open symbols) the induction of mutant dynamin: HeLa dynK44A cells were grown in the presence or the absence of tetracycline (Tet) for 2 days. The cells were then washed, and ^125^I-toxins were added. The cells were incubated at 37 °C for 0 to 120 min, and bound and endocytosed toxins were quantified as described in Section 4.2.2. (**b**) Endocytosis of ^125^I-labeled transferrin in HeLa dynK44A cells with (filled bar) and without (open bar) the induction of mutant dynamin: endocytosed transferrin was quantified after 5 min of internalization. (**c**) Degradation of ebulin l and ricin in HeLa dynK44A cells: the cells were grown in the presence (open bar) or the absence (filled bar) of tetracycline for 2 days. The cells were then transferred to a HEPES-containing medium and preincubated without or with (+Baf) bafilomycin A1 (1 mM) for 30 min at 37 °C. ^125^I-toxins were then added, and 20 min later, the surface-bound toxins were removed with a 0.1 M lactose solution at 37 °C. The incubation was continued in the presence or in the absence of bafilomycin A1, and after 2 h, further incubation toxin degradation was measured as described in Section 4.2.2. The data represent the mean ± SD of two experiments. (**d**) Uptake of ^125^I-transferrin (△),^125^I-ebulin l (●), and ^125^I-ricin (☐) by HeLa dynK44A cells. The cells grown in the presence of tetracycline were incubated for 5 min at 37 °C in HEPES medium, pH 5.5, with increasing concentrations of acetic acid. ^125^I-transferrin or ^125^I-toxins were then added as described above, and after 5 and 20 min of incubation, the amount of endocytosed proteins was measured as in (a). The data represent the mean ± SD of two experiments performed in duplicate.

We next studied the internalization of ebulin l and ricin both in cells where the mutant dynamin was overexpressed and in cells where its expression was repressed by the presence of tetracycline. Control experiments showed that the endocytosis of ^125^I-transferrin, which occurs from coated pits [28], was inhibited by more than 90% by overexpression of the mutant dynamin (Figure 6b). By contrast, ^125^I-ebulin l and ^125^I-ricin uptake were unchanged in cells expressing the mutant dynamin (Figure 6a). Approximately 25% of total cell-associated ^125^I-ebulin l and 15% of total cell-associated ^125^I-ricin were internalized into HeLa dynK44A cells, with and without the induction of mutant dynamin, during 30 min of incubation at 37 °C. To study whether the transport of ebulin l to lysosomes was affected by the expression of mutant dynamin, toxin degradation was measured. As shown in Figure 6c, essentially the same degradation rates were obtained whether the mutant dynamin was expressed by the removal of tetracycline or not. The percentage of ebulin l released by cells in the TCA-soluble fraction was higher (34%) than that of ricin (9%) (Figure 6c). Bafilomycin A1 inhibited toxin degradation, indicating that the process took place in a low-pH compartment (Figure 6c). Moreover, recycling of ebulin l and ricin was not affected by the overexpression of dyn K44A (data not shown) and the values obtained were comparable to those observed in COS cells (Figure 2b). Therefore, the data indicate that the endocytic uptake of ebulin l and ricin, at least in HeLa cells with dynK44A overexpression, does not occur by clathrin-coated pits or caveolae. According to this, it has been shown earlier that ricin endocytosis continued to the same level after dynK44A expression [35]. The cells therefore seem then to be able to upregulate clathrin-independent endocytosis under conditions where a prolonged inhibition of clathrin-dependent endocytosis takes place [35,38].

Our results indicated that, after a prolonged inhibition of clathrin-dependent endocytosis in HeLa dyn K44A expressing the mutant dynamin, ebulin l endocytosis continues (Figure 6a), while in COS cells, there was a 50% reduction of the endocytic uptake of ebulin l when endocytosis from coated pits was acutely inhibited by acidification of the cytosol (Figure 3). To test if this is also the case in HeLa dynK44A cells, the uptake of ^125^I-labeled ebulin l and ricin was measured in cells grown with tetracycline that had been acidified by incubation with acetic acid. Figure 6d shows that, in these cells, the uptake of ebulin l was reduced by about 45% after acidification of the cytosol. The uptake of transferrin was reduced by more than 95% under the same conditions. These results therefore indicate that there is no difference between COS cells and HeLa cells in the way they endocytose ebulin l and ricin. Thus, prolonged inhibition of clathrin-dependent uptake in cells expressing the mutant dynamin can induce an increase in clathrin-independent endocytosis [35,38] while acute inhibition of clathrin-dependent endocytosis by acidifying the cytosol cannot. Endocytosis by mechanisms not involving clathrin-coated pits has been shown for several bacterial toxins such as tetanus toxin, cholera toxin, and plant RIPs such as ricin, lanceolin, and stenodactylin [35,39,40].

### 2.3. Effect of DynK44A Overexpression and Cytosol Acidification on Ebulin l and Ricin Cytotoxicity

**Figure 7:**
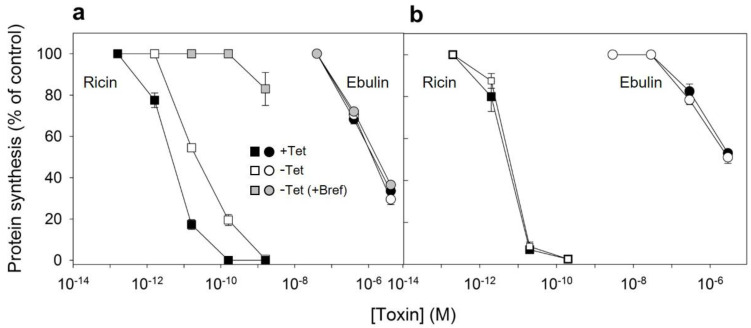
Ability of ebulin l and ricin to inhibit protein synthesis in HeLa dynK44A (**a**) and COS cells (**b**): dynK44A cells were grown with (closed symbols) and without (open symbols) tetracycline (Tet) for 2 days. Then, the cells were incubated with different concentrations of ebulin l (circles) and ricin (squares) for 18 h, and protein synthesis was measured as indicated in Section 4.2.4. To investigate the effect of brefeldin A (grey symbols) on protein synthesis of K44A cells grown without tetracycline, the cells were preincubated for 1 h with brefeldin A and then incubated with different concentrations of ebulin l and ricin for 18 h, and protein synthesis was measured. The data represent the mean ± SD of two experiments performed in duplicate. (**b**) The toxic effect of ebulin l (circles) or ricin (squares) endocytosed at normal (open symbols) and acidic internal pH (closed symbols): COS cells were incubated for 30 min at 37 °C in HEPES medium, pH 7, with and without 25 mM of NH_4_Cl. The medium was removed, and a solution containing 0.14 M KCl, 2 mM CaCl_2_, 1 mM amiloride, 1 mM MgCl_2_, and 20 mM HEPES, pH 7.0, was added. After 5 min of incubation at 37 °C, increasing concentrations of ebulin or ricin were added and, after 20 min of further incubation, a growth medium containing 0.1 M lactose was added. The cells were then incubated for 18 h, and protein synthesis was measured. The data represent the mean ± SD of two experiments performed in duplicate.

After endocytosis and transport to the Golgi apparatus and the endoplasmic reticulum, a small number of ricin molecules reach the cytosol, inhibiting protein synthesis. It has been shown earlier that cells overexpressing dynK44A were more resistant to ricin than dynK44A cells expressing endogenous dynamin [35]. The expression of mutant dynamin inhibits transport of endocytosed ricin to the Golgi apparatus, and this transport is important for ricin intoxication [35]. To investigate whether the overexpression of dynK44A changes the ability of ebulin l to intoxicate cells, we measured protein synthesis 18 h after the addition of increasing concentrations of ebulin l or ricin to dynK44A cells grown with and without tetracycline. As shown in Figure 7a, the toxicity of ebulin l was not affected by the expression of mutant dynamin while, as expected, it protected the cells against ricin. Under these conditions, uninduced HeLa dynK44A cells were about 4.8 times more sensitive to ricin that dynK44A-induced cells. In addition, we also studied whether ebulin l and ricin internalized by the clathrin- and caveolae-independent pathway in these cells must be transported through the Golgi apparatus to inhibit protein synthesis. In these experiments, HeLa cells with mutant dynamin were pretreated with brefeldin A, which disrupts the Golgi apparatus, and protein synthesis was measured 18 h later. As shown in Figure 7a and consistent with our previous observations (Figure 5), brefeldin A did not protect cells from the inhibition of protein synthesis by ebulin l but the cells were completely protected against ricin. These data clearly indicate that transport through the Golgi is not required for ebulin l intoxication.

Since the endocytic uptake of ebulin l and ricin in COS cells was reduced by 50% by acidification of the cytosol (Figure 3), we decided to study if toxin internalized under such conditions (by clathrin-independent endocytosis) can intoxicate cells. In these experiments, we used the NH_4_Cl pre-pulse method to acidify the cytosol. Ebulin l or ricin were added to COS cells, and endocytosis was allowed to proceed for 20 min. Then, the cell surface-bound toxins were removed with a medium containing 0.1 M lactose and the cells were incubated 18 h in normal medium to allow the internalized toxin to intoxicate the cells. After that, the ability of the cells to incorporate [^3^H] leucine was measured. The data in Figure 7b show that protein synthesis in cells treated in this way was inhibited by ebulin l and ricin to the same extent as in control cells, where endocytic uptake of the RIPs occurred at normal internal pH. The data suggest that, in COS cells, the endocytosis of ebulin l that leads to intoxication of cells takes place predominantly from clathrin-independent mechanisms, and thus, there is no apparent role for clathrin in productive intracellular transport.

### 2.4. Ebulin l Induces Apoptosis in COS Cells

**Figure 8:**
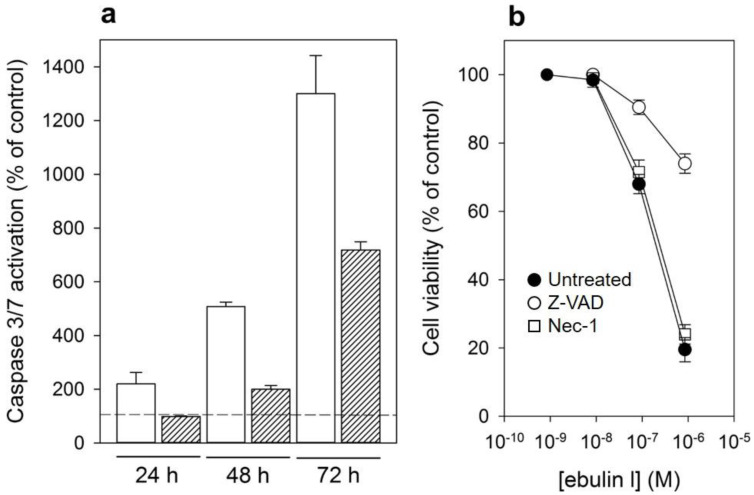
(**a**) Caspase-3/7 activation in COS cells treated with 10^−6^ M (empty bar) or 10^−7^ M (filled bar) ebulin l for 24, 48, and 72 h: activity is expressed as the percentage of control values obtained from cells grown in the absence of ebulin l (dashed line). The data represent the mean ± SD of two experiments performed in duplicate. (**b**) Effect of Z-VAD and Necrostatin (Nec-1) on cytotoxicity of ebulin l on COS cells: the cells were preincubated with Z-VAD or Necrostatin for 3 h or left untreated, and then, different concentrations of ebulin l were added and the cells were incubated for 48 h. Cell viability was assessed by a colorimetric assay as indicated in Section 4.2.4. The data represent the mean ± SD of two experiments performed in triplicate. Symbols: ●, untreated; ○, +Z-VAD; and ☐, +Nec-1.

In addition to rRNA damage, RIPs are capable of inducing cell death by apoptosis [4]. We therefore decided to investigate the death pathways involved in the cytotoxicity of ebulin l in COS cells. Cells treated with ebulin l exhibited the morphological features characteristic of apoptosis such as cell rounding and blebbing (data not shown). To demonstrate the involvement of caspase-dependent apoptosis, caspase-3/7 activation was measured in COS cells exposed to 10^−6^ and 10^−7^ M ebulin l for 24, 48, and 72 h. As shown in Figure 8a, a time- and dose-dependent activation of effector caspases was observed in COS cells. Caspase activity seems to be significantly induced after treating the cells with 10^−6^ M ebulin l for 24 h, and at that concentration, protein synthesis was inhibited by 50% after 18 h (Figure 5). Thus, protein synthesis inhibition seems to be an earlier event than apoptosis in these cells and suggests that apoptosis might be a consequence of the ribotoxic stress induced by the A chain after entry into the cytosol. However, we cannot rule out the possibility that both processes run in parallel. To evaluate the role of the different cytotoxic mechanisms induced by ebulin l, COS cells were pretreated with two inhibitors, the pan-caspase inhibitor Z-VAD, which irreversibly binds to the catalytic site of caspases and was used to selectively inhibit the apoptotic pathway, and the inhibitor of necroptosis, Necrostatin. COS cells were pretreated and maintained in 100 μM Z-VAD, and the cell viability was determined for different ebulin l concentrations. As shown in Figure 8b, caspase inhibition by Z-VAD largely prevented the cytotoxicity of ebulin l after 48 h. At a concentration of 10^−6^ M, viability increased from 20% to 72% in the presence of Z-VAD. In contrast, the necroptosis inhibitor Necrostatin did not rescue ebulin l-induced cell death. (Figure 8b). Therefore, these data indicate that apoptosis is the predominant pattern of cell death induced by ebulin l.

## 3. Conclusions

This work contributes to elucidating the mechanisms involved in the endocytosis and intracellular transport of the plant toxin ebulin l. Our results demonstrate that ebulin l has a lower number of binding receptors than ricin in COS and HeLa dynK44A cells. This may be due to a change in the structural disposition of the 2γ-subdomain of the ebulin B chain, which limits its ability to bind galactosides. Following binding, ebulin l is internalized by both clathrin-dependent and -independent mechanisms. A short time after internalization, ebulin l is localized to early endosomes and later to lysosomes but apparently not to the Golgi apparatus. The ebulin l molecules that lead to intoxication of cells are internalized via clathrin-independent mechanisms. Moreover, the cytotoxic effect of ebulin l occurs independently of low endosomal pH and does not require transport of the toxin through the Golgi apparatus. Moreover, ebulin l induces a caspase-dependent apoptosis as the predominant cell death mechanism. Importantly, toxins have a potential as therapeutic agents if the toxicity can be targeted to malignant cells. The low unspecific toxicity of ebulin l together with its strong anti-ribosomal activity and induction of apoptosis make it an excellent candidate as a toxic moiety in the construction of immunotoxins and conjugates directed against specific targets. Knowledge of the mechanisms of transport and action of the toxin is essential in achieving this goal.

## 4. Materials and Methods

### 4.1. Reagents and Cells

The sources of the chemicals and cells were described previously [16,29,34,35,41]. Particular details are given in the Supplementary File S1.

### 4.2. Methods

Particular experimental details are given in the Supplementary File S1.

#### 4.2.1. Binding of ^125^I-Labeled Toxins to Cells and Crosslinking of ^125^I-ebulin l to Membrane Receptors

Confluent cells were washed twice with ice-cold HEPES medium and incubated at 4 °C for 15 min before increasing concentrations of ebulin l or ricin were added. The cells were incubated for 1 h with the toxins and then washed five times with ice-cold phosphate buffered saline (PBS). The radioactivity was measured after dissolving the cells in 0.1 M KOH. Nonspecific binding was estimated by the incubation of cells in the presence of 0.1 M lactose. Receptor dissociation constants and the number of binding sites were estimated by the Scatchard method [42].

Crosslinking of bound ^125^I-ebulin l to cells was carried out with disuccinimidyl suberate [43].

#### 4.2.2. Measurements of Endocytosis, Recycling, and Degradation

Endocytosis of transferrin was measured after incubation for 5 min with transferrin (100 ng/mL, labeled with ^125^I) [35]. Internalized toxin (400 ng/mL) was measured as the amount of ^125^I-labeled toxin that was not removed after incubating the cells with a 0.1 M lactose solution for 5 min at 37 °C [44]. Recycling and degradation of toxins were measured as previously described [44].

Endocytosis in cells with acidified cytosol by NH_4_Cl pre-pulsing or by incubation with acetic acid was assessed as previously described [27]. Cell-bound and endocytosed proteins were measured after 20 min of incubation for ^125^I-ebulin and ^125^I-ricin and 5 min for ^125^I-transferrin.

#### 4.2.3. Immunofluorescence Microscopy

Binding of CY3-ebulin l and double-staining experiments were carried out as previously described [43]. The coverslips were examined with a Zeiss LSM 510 META confocal microscope (Carl Zeiss, Jena, Germany). Colocalization analysis were performed using Coloc2 (version 3.0.0) in Fiji (ImageJ 1.53c) (http://fiji.sc/wiki/index.php/Fiji).

#### 4.2.4. Other Measurements

Protein synthesis was measured as previously described [35]. Cell viability was determined with a colorimetric assay based on cleavage of the tetrazolium salt WST-1 to formazan and the caspase-3/7 activity was assessed by the luminescent assay Caspase-GloTM 3/7 [16].

## Data Availability

Data are available upon request; please contact the contributing authors.

## Supplementary Materials

The following are available online at https://www.mdpi.com/2072-6651/13/2/102/s1, Figure S1: Toxin binding and Scatchard plots, File S1: Description of the materials and methods.
